# The Relationship between Metabolic Syndrome and Plasma Metals Modified by EGFR and TNF-α Gene Polymorphisms

**DOI:** 10.3390/toxics9090225

**Published:** 2021-09-16

**Authors:** Tzu-Hua Chen, Wei-Shyang Kung, Hung-Yu Sun, Joh-Jong Huang, Jia-Yi Lu, Kuei-Hau Luo, Hung-Yi Chuang

**Affiliations:** 1 Department of Public Health, College of Health Sciences, Kaohsiung Medical University, Kaohsiung 80708, Taiwan; 980264@kmuh.org.tw (T.-H.C.); u105570008@kmu.edu.tw (J.-Y.L.); 2 Department of Family Medicine, Kaohsiung Municipal Ta-Tung Hospital, Kaohsiung 80145, Taiwan; 3 Department of Family Medicine, Kaohsiung Medical University Hospital, Kaohsiung 80708, Taiwan; 1030578@kmuh.org.tw; 4 Department of Pediatrics, Chien Shin Hospital, Kaohsiung 80143, Taiwan; u104862002@kmu.edu.tw; 5 Department of Family Medicine, Changhua Christian Hospital, Changhua 50006, Taiwan; 144107@cch.org.tw; 6 Graduate Institute of Medicine, College of Medicine, Kaohsiung Medical University, Kaohsiung 80708, Taiwan; u107800007@kmu.edu.tw; 7 Department of Environmental and Occupational Medicine, Kaohsiung Medical University Hospital, Kaohsiung 80708, Taiwan; 8 Ph.D. Program in Environmental and Occupational Medicine, Research Center for Environmental Medicine, College of Medicine, Kaohsiung Medical University, Kaohsiung 80708, Taiwan

**Keywords:** metabolic disorders, metabolic syndrome, epidermal growth factor receptor (EGFR), TNF-α, single nucleotide polymorphism (SNP), metals, inflammation, insulin resistance, Taiwan biobank

## Abstract

With the escalating global prevalence of metabolic syndrome (MetS), it is crucial to detect the high-risk population early and to prevent chronic diseases. Exposure to various metals has been indicated to promote MetS, but the findings were controversial, and the effect of genetic modification was not considered. Epidermal growth factor receptor (EGFR) was proposed to be involved in the pathway of metabolic disorders, and tumor necrotic factor-α (TNF-α) was regarded as an early inflammatory biomarker for MetS. This research aimed to analyze the impact of EGFR and TNF-α gene polymorphisms on the prevalence of MetS under environmental or occupational exposure to metals. We gathered data from 376 metal industrial workers and 639 non-metal workers, including physical parameters, biochemical data, and plasma concentrations of six metals. According to the genomic database of Taiwan Biobank, 23 single nucleotide polymorphisms (SNPs) on EGFR gene and 6 SNPs on TNF-α gene were incorporated in our research. We applied multivariable logistic regression to analyze the probability of MetS with various SNPs and metals. Our study revealed some susceptible and protective EGFR and TNF-α genotypes under excessive exposure to cobalt, zinc, selenium, and lead. Thus, we remind the high-risk population of taking measures to prevent MetS.

## 1. Introduction

Metabolic syndrome (MetS), a prodromal stage of cardiovascular disease (CVD) and type 2 diabetes, has attracted wide public attention because it may contribute to all-cause mortality [1,2]. The global prevalence of MetS is estimated to be 25% and still escalating [3,4]. Therefore, it is imperative to identify the high-risk population and develop preventive strategies to reduce the health burden of chronic diseases.

In addition to dietary habits, exercise, and genetic inheritance [5], some research studies have indicated the associations between metal exposure and MetS, but the findings were controversial [6,7]. A Korean population-based study suggested that elevated blood lead level was associated with a higher prevalence of MetS [8]; on the contrary, an inverse association was observed between blood lead and MetS in the residents of the US [6]. Adequate selenium supplement might be beneficial [9], while some other studies supposed that selenium may contribute to MetS [10]. The complex interactions between environmental and genetic factors promote MetS, and many genetic variants were reported to be involved in the pathophysiology of MetS [11,12,13]. Nevertheless, the literature about genes influencing the risk of MetS under metal exposure is still limited.

The epidermal growth factor receptor (EGFR) is a kind of receptor tyrosine kinase. Upon ligand binding, EGFR activation initiates multiple signaling pathways, which regulate the cellular processes, such as metabolism, proliferation, and migration [14]. EGFR is expressed in the vascular wall and myocardium, and EGFR transactivation may contribute to arterial hypertension due to abnormal regulation of vascular tone [15,16]. In animal studies, EGFR may play a vital role in lipid metabolism in adult male mice [17], and EGFR inhibitors had effects on reducing serum lipid levels and hepatic steatosis in high-fat-diet-induced obese mice [18,19]. Furthermore, EGFR blockade was observed to reduce blood glucose and ameliorate insulin resistance [20]. EGFR-mediated pathway in metabolic disorders is fascinating, and EGFR inhibitors may be promising therapeutic targets for cardiovascular and metabolic diseases [18,19].

Tumor necrotic factor-α (TNF-α), a kind of inflammatory mediator, was found to be elevated in obese people [21,22]. TNF-α overexpression plays a crucial role in the development of insulin resistance, which is an important component of MetS [23]. A systematic review suggested that TNF-α could be an early biomarker to detect MetS [24]. Some studies have demonstrated that TNF-α gene polymorphisms were associated with the susceptibility of MetS, but the results were inconsistent in different ethnic groups [25]. Making an effort to elucidate the interactions of TNF-α genetic variants and environment was considered as a potential method to improve MetS [26]. Nevertheless, the relationship between TNF-α gene polymorphisms and MetS under the influence of metal exposure is not clear.

Humans are likely to be exposed to a variety of metals through industrial, agricultural, or technological applications in their working and living environment [27]. Among the most common types of exposure, arsenic (As) and lead (Pb) are regarded as harmful heavy metals, while cobalt (Co), copper (Cu), zinc (Zn), and selenium (Se) are essential trace elements but may cause a threat to health beyond the optimum concentration [28,29]. The aim of our study is to investigate the relationship between metal exposure and the prevalence of MetS under the modification of EGFR and TNF-α gene polymorphisms.

## 2. Materials and Methods

### 2.1. Study Population and Definition of Metabolic Syndrome

The study subjects were divided into two groups, metal industrial workers and non-metal industrial workers, and their ages ranged from 18 to 65 years old. A total of 376 metal workers who received regular health examinations in Kaohsiung Medical University Hospital were qualified to take part in this research. With regard to non-metal workers, 241 subjects were the applicants undergoing health checkup packages in the hospital, and 398 subjects were participants from Taiwan Biobank (TWB). TWB, the largest official biobank in Taiwan, was established to record the genomic databases and lifestyles of the Taiwanese population [30]. All participants received anthropometric measurements, physical examinations, and venous blood sampling. The questionnaires of face-to-face interviews consisted of medical history, medication record, tobacco and alcohol habits, and occupation. The subjects with cancer history were excluded from the study. All procedures were approved by the Kaohsiung Medical University Hospital Institutional Review Board (approval number: KMUHIRB-E(I)-20150259), and all individuals signed the approved informed consent form. 

Based on the National Cholesterol Education Program–Adult Treatment Panel III (NCEP–ATP III) [31], we used the modified criteria by the Taiwan Bureau of Health Promotion to evaluate MetS [32]. The individuals with at least three of the following five abnormalities are diagnosed with MetS: (1) waist circumference (WC) ≥ 90 cm in men and ≥ 80 cm in women; (2) systolic blood pressure (SBP) ≥ 130 mmHg or diastolic blood pressure (DBP) ≥ 85 mmHg, or under treatment for hypertension; (3) fasting sugar ≥ 100 mg/dL or under treatment for diabetes; (4) high-density lipoprotein cholesterol (HDL–C) < 40 mg/dL in men and <50 mg/dL in women; (5) triglyceride (TG) ≥ 150 mg/dL.

### 2.2. Analyses of Plasma Metals

Plasma concentrations of all metals and elements were analyzed by Inductively Coupled Plasma Mass Spectrometry (ICPMS, Thermo Scientific XSERIES 2) at the laboratory in Kaohsiung Medical University. Radio Corporation of America (RCA) clean was used on all equipment in the laboratory. For sample preparation, 1% HNO_3_ was added into plasma samples to make 1:10 dilution and then left for 10 minutes. To check high linearity, ICP-MS calibration standard solution (Accu Standard, MES-04-1) was diluted to 0.1, 0.2, 0.5, 1, 2, 5, 10, 20, 50, 100, 200, 500, 1000, 2000, 3000 μg/L to estimate the calibration curve, and each element was consistent with the curve with high correlation coefficient (r > 0.995). We checked plasma concentrations of Co, Cu, Zn, Se, As, and Pb. Before analyzing the unknown concentrations, we conducted quality assurance (QA) and quality control (QC) to ensure precision and accuracy. QA was used to analyze standard reference materials (SRMs). To ensure the consistency of laboratory tests, we took random SRMs to conduct repeated analysis, and each result had to fit the curve between 90% and 110%. QC was to make sure the stability of the system by triple-testing SRMs, whose coefficient of variance (CV) should be less than 3%.

### 2.3. Genotyping

Genotyping of single-nucleotide polymorphisms (SNPs) was performed using the custom TWB chips and run on the Axiom Genome-Wide Array Plate System (Affymetrix, Santa Clara, CA, USA). TWB1 array was designed for Taiwan’s Han Chinese and released in April 2013. Furthermore, the TWB2 array released in August 2018 was based on the experience of TWB1 use and designed for further clinical use. There were approximately 653k autosomal SNPs and 752k SNPs that could be genotyped in TWB1 and TWB2 arrays, respectively. There were about 105k overlapping SNPs in two TWB arrays [33].

**Figure 1:**
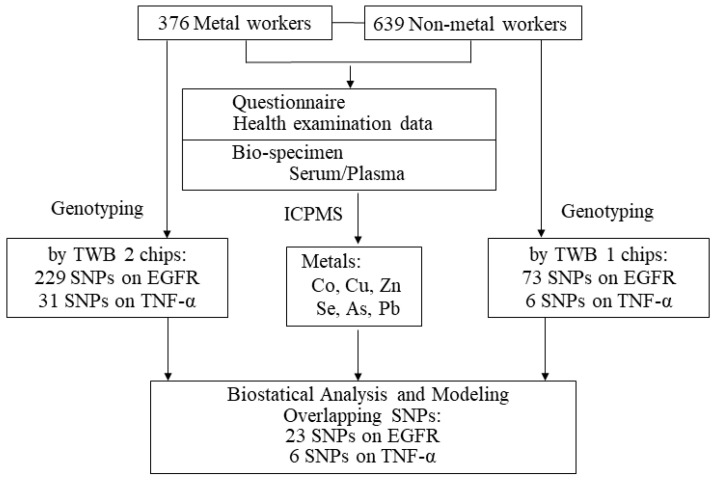
The study protocol.

The individuals of non-metal worker group were genotyped using TWB1 chips, while those of metal worker groups were genotyped using TWB2 chips. Regarding QC in the genetic study, we used PLINK 1.9 to calculate the genome-wide identity by descent (IBD) to ensure the unrelatedness of all DNA samples, and we excluded the individuals with IBD > 0.1875 [34,35]. We also excluded the SNPs with genotyping rate < 95% and Hardy–Weinberg test *p*-values < 10^−6^ [35,36]. Moreover, we used (N-O)/N to calculate mean heterozygosity, where N was the number of non-missing genotypes and O was the observed number of homozygous genotypes for a given subject. Individuals with more than 3 standard deviations from mean heterozygosity were excluded due to the possibility of DNA sample contamination or inbreeding [35]. After the QC process, TWB1 chips revealed 73 SNPs on EGFR gene and 6 SNPs on TNF-α gene, and TWB2 chips revealed 229 SNPs on EGFR gene and 31 SNPs on TNF-α gene (Supplementary Tables S1 and S2). Between these two arrays, 23 SNPs on EGFR gene and 6 SNPs on TNF-α gene were overlapped, and thus a total of 29 SNPs were kept in our analysis. The study protocol was summarized as Figure 1. 

### 2.4. Statistical Analyses

We used the Chi-square test and t-test for analyzing between-group differences. Multivariable logistic regression analysis was applied to calculate the odds ratio (OR) and 95% confidence interval (CI) of MetS with various SNPs and plasma metal concentrations. Age, gender, and the habit of smoking and drinking alcohol were incorporated as covariates.

Initially, to examine whether gene polymorphisms was significantly associated with MetS, we regressed MetS on each of the 29 SNPs with adjustment for covariates:

logit [p(MetS)/1 − p(MetS)] = β_0_ + β_SNP*,i*_ SNP*_i_* + β_c_ Covariates + ε, *i* = 1,...,29, (1)

where SNP*_i_* is the number of minor alleles at the *i*th SNP (0, 1, or 2) and ε is the error term. By testing H_0_: β_SNP*,i*_ = 0 versus H_1_: β_SNP*,i*_ ≠ 0, we obtained ORs and 95% CIs for associations between the *i*th SNP and MetS.

We also explored the relationship between 6 plasma metal concentrations and MetS:

logit [p(MetS)/1 − p(MetS)] = β_0_ + β_M,*j*_ M*_j_* + β_c_ Covariates + ε, *j* = 1,...,6, (2)

where M is the plasma concentration of Co, Cu, Zn, Se, As, and Pb, respectively.

Then, we regressed MetS on every metal and every SNP, and a total of 174 logistic regression models were summarized below:

logit [p(MetS)/1 − p(MetS)] = β_0_ + β_M,*j*_ M_j_ + β_SNP,1*i*_ SNP_1*i*_ + β_SNP,2*i*_ SNP_2*i*_ + β_c_ Covariates + ε, (3)

where two dummy variables were used in the genotypes. We set major allele homozygous genotypes as reference. We set SNP_1*i*_ = 1 for heterozygous genotypes and SNP_1*i*_ = 0, otherwise. Similarly, we set SNP_2*i*_ = 1 for minor allele homozygous genotypes and SNP_2*i*_ = 0, otherwise.

Finally, we considered the interaction between metals and SNPs, and we built 174 logistic regression models:

logit [p(MetS)/1 − p(MetS)] = β_0_ + β_M,*j*_ M_j_ + β_SNP,1*i*_ SNP_1*i*_ + β_SNP,2*i*_ SNP_2*i*_ + β_int,1*i*_ M_j_ × SNP_1*i*_ +β_int,2*i*_ M_j_ × SNP_2*i*_ + β_c_ Covariates + ε. (4)

The analyses were executed by SAS package (version 9.4; SAS Institute, Cary, NC, USA), and a two-tailed *p*-value < 0.05 indicated statistical significance. Moreover, we also tried to adjust for multiple testing by using the Bonferroni correction. The *p*-values of *p* < 0.05/23 = 0.002 in EGFR SNPs, and *p* < 0.05/6 = 0.008 in TNF-α SNPs were considered more stringent.

## 3. Results

Table 1 compares the demographic characteristics, physical and biochemical data, and plasma metal concentrations among the participants with and without MetS. The percentages of metal industrial workers, male subjects, and smokers were higher in the individuals with MetS. Those with MetS had older age and higher body mass index (BMI), WC, SBP, DBP, fasting sugar, TG, total cholesterol, uric acid, alanine aminotransferase (ALT), and creatinine, whereas they had lower HDL-C. Moreover, those with MetS had higher plasma concentration of Cu, Zn, Se, and Pb.

**Figure 2:**
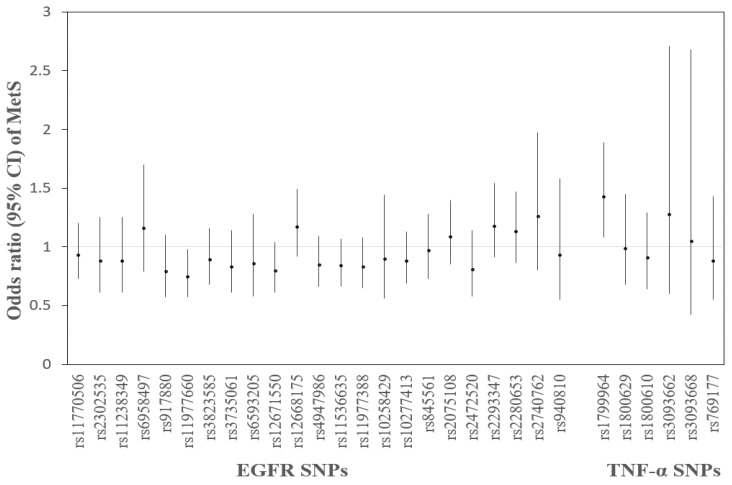
Odds ratios (ORs) and 95% confidence intervals (CIs) of metabolic syndrome (MetS) with 29 SNPs, respectively. Adjusted for age, gender, smoking, and drinking alcohol. (rs1799964, rs1800629, rs1800610, rs3093662, rs3093668, and rs769177 are TNF-α SNPs, and the others are EGFR SNPs.).

According to Equation (1), the prevalence of MetS with each SNP had a decrease in rs11977660 T > C (OR = 0.75, 95% CI: 0.57, 0.98) and an elevation in rs1799964 T > C (OR = 1.43, 95% CI: 1.08, 1.89) (Figure 2).

**Figure 3:**
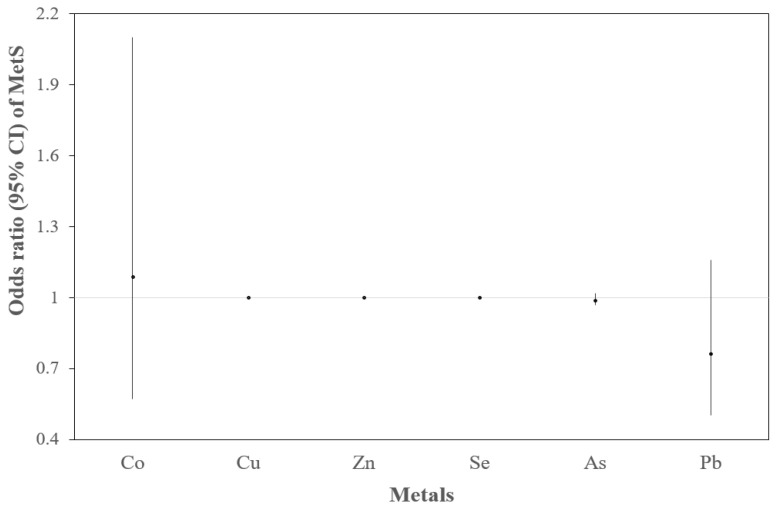
ORs and 95% CIs of MetS with 6 metals, respectively. Adjusted for age, gender, smoking, and drinking alcohol. The OR of MetS with Cu was 1.001 (95% CI: 1.000, 1.001). The prevalence of MetS had no association with Zn, Se, As, and the ORs were small (OR = 1.000, 1.001, 0.992, respectively).

According to Equation (2), the prevalence of MetS with each metal had an increase in Cu only (OR = 1.001, 95% CI: 1.000, 1.001) (Figure 3).

**Figure 4:**
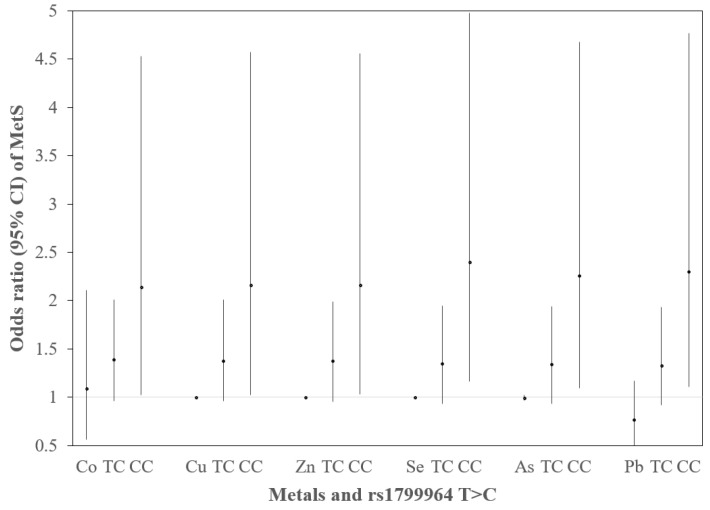
ORs (95% CIs) of MetS for rs1799964 T>C by metals. The logistic regression model was built as logit [p(MetS)/1 − p(MetS)] = β_0_ + β_M,*j*_ M*_j_* + β_SNP,1*i*_ SNP_1*i*_ + β_SNP,2*i*_ SNP_2*i*_ + β_c_ Covariates + ε. Adjusted covariates included age, gender, smoking, and drinking alcohol. The prevalence of MetS with metals was increased in Cu only (OR = 1.001, 95% CI: 1.000, 1.001). Genotype CC was associated with an elevated risk of MetS, and the OR was 2.14 (95% CI: 1.02, 4.53) adjusting for Co, OR = 2.16 (95% CI: 1.02, 4.57) adjusting for Cu, OR = 2.16 (95% CI: 1.03, 4.56) adjusting for Zn, OR = 2.40 (95% CI: 1.16, 4.98) adjusting for Se, OR = 2.26 (95% CI: 1.09, 4.68) adjusting for As, and OR = 2.30 (95% CI: 1.11, 4.77) adjusting for Pb.

According to Equation (3), the prevalence of MetS had an association with metals and rs1799964 T > C (Figure 4). The prevalence of MetS with metals showed an increase in Cu only (OR = 1.001, 95% CI: 1.000, 1.001). Compared with genotype TT, genotype CC was associated with an elevated risk of MetS, and the OR was 2.14 (95% CI: 1.02, 4.53) adjusting for Co, 2.16 (95% CI: 1.02, 4.57) adjusting for Cu, 2.16 (95% CI: 1.03, 4.56) adjusting for Zn, 2.40 (95% CI: 1.16, 4.98) adjusting for Se, 2.26 (95% CI: 1.09, 4.68) adjusting for As, and 2.30 (95% CI: 1.11, 4.77) adjusting for Pb.

**Figure 5:**
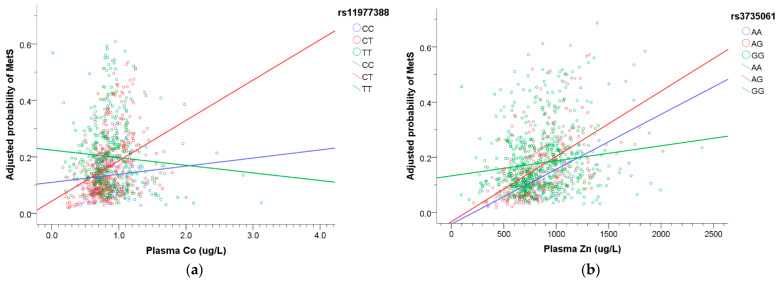

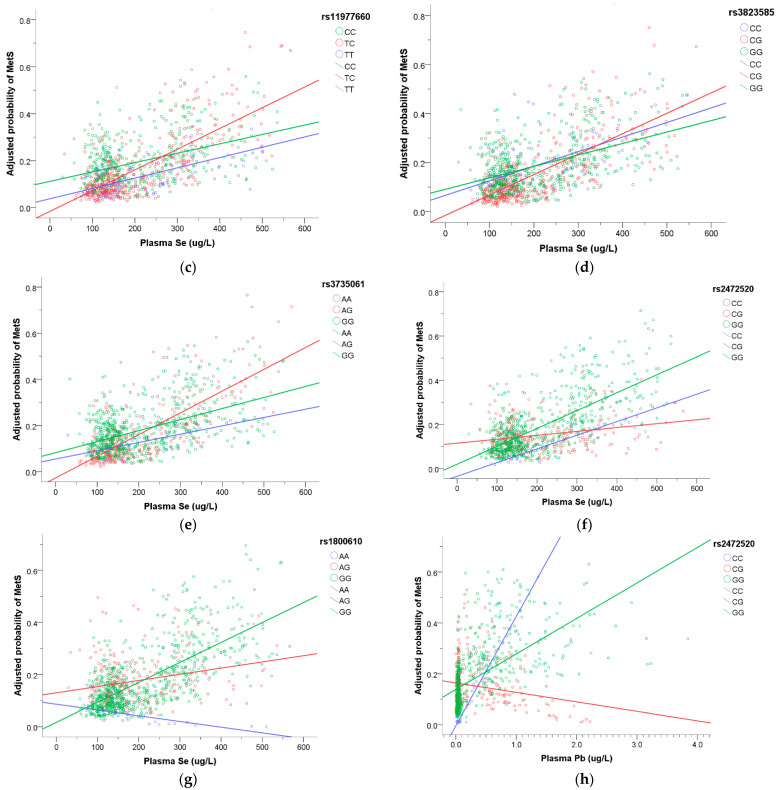
The association of plasma metals with the risk of MetS modified by SNPs. The regression model was built as logit [p(MetS)/1 − p(MetS)] = β_0_ + β_M,*j*_ M*_j_* + β_SNP,1*i*_ SNP_1*i*_ + β_SNP,2*i*_ SNP_2*i*_ + β_int,1*i*_ M*_j_* × SNP_1*i*_ +β_int,2*i*_ M*_j_* × SNP_2*i*_ + β_c_ Covariates + ε. Adjusted covariates included age, gender, smoking, and drinking alcohol. (**a**) The risk of MetS was much higher in rs11977388 CT genotype with increasing plasma Co. (**b**) Increasing plasma Zn with higher risk of MetS, especially in rs3735061 AG genotype. (**c**) Increasing plasma Se with higher risk of MetS, especially in rs11977660 TC genotype. (**d**) Increasing plasma Se with higher risk of MetS, especially in rs3823585 CG genotype. (**e**) Increasing plasma Se with higher risk of MetS, especially in rs3735061 AG genotype. (**f**) Increasing plasma Se with higher risk of MetS but the least elevated risk in rs2472520 CG genotype. (**g**) The risk of MetS reduced in rs1800610 AA genotype with increasing plasma Se. (**h**) The risk of MetS decreased in rs2472520 CG genotype with increasing plasma Pb.

According to Equation (4), we plotted Figure 5 to express the interactions between metals and SNPs that influence the prevalence of MetS. When the plasma Co concentration increased, there was an elevated prevalence of MetS in rs11977388 CT genotype (Figure 5a). When the plasma Zn level increased, the prevalence of MetS increased, especially in rs3735061 AG genotype (Figure 5b). When the plasma Se concentration increased, the risk of MetS increased the most in rs11977660 TC, rs3823585 CG, and rs3735061 AG genotype (Figure 5c–e), while the prevalence of MetS had the least increase in rs2472520 CG genotype (Figure 5f) and decreased in rs1800610 AA genotype (Figure 5g). When the plasma Pb level increased, the risk of MetS decreased in rs2472520 CG genotype (Figure 5h). However, only rs2472520 CG x plasma Pb remained significant after Bonferroni correction (*p* < 0.05/23 = 0.002).

## 4. Discussion

Some studies have demonstrated that metal exposures are related to MetS, but the results were inconsistent [6,7]. Moreover, the effect modified by genetic polymorphisms was scarcely mentioned. In this cross-sectional study from metal industrial workers and the participants of TWB and health examination, we firstly assessed the relationship between MetS risk and plasma metals (Co, Cu, Zn, As, Se, and Pb) and genetic polymorphisms (EGFR and TNF-α) separately. Then, we determined whether EGFR and TNF-α SNPs modified the associations of plasma metal concentrations with MetS risk.

Our research revealed that higher plasma Cu concentration may be associated with higher risk of MetS. Although Cu is an essential trace element for humans, excessive amounts of Cu may increase the formation of reactive oxygen species (ROS), inducing cellular toxicity [37]. Similar to our finding, elevated urinary Cu concentration was reported to be associated with MetS [7,28]. With regard to the mechanism, Ma et al. demonstrated that there was a positive correlation between urinary Cu and plasma C-reactive protein (CRP) [28] and that CRP was associated with MetS [38]. Furthermore, our previous research showed that blood Cu concentration was positively associated with serum TNF-α [39]. We proposed that the association of Cu exposure with MetS may be on the basis of systemic inflammation.

Our results showed that EGFR rs11977660 T > C and TNF-α rs1799964 T > C were associated with MetS. EGFR rs11977660 TT and TC genotypes were reported to increase the risk of endometriosis [40]; on the other hand, they were not associated with renal cancer [41] and lung adenocarcinoma [42]. Our result showed that the additive model of rs11977660 T > C was associated with a reduced MetS risk, and the individuals carrying TC genotype had an elevated MetS risk with increasing plasma Se levels. We supposed that EGFR rs11977660 T > C may play different roles in various diseases, and the effect may be influenced by the interaction with environment.

TNF-α gene polymorphisms are associated with many diseases, such as infection, autoimmune diseases, rejection in organ transplantation, and so on [43]. Our results showed that TNF-α rs1799964 CC genotype was associated with an elevated risk of MetS either without or with adjustment for metals. This finding was in line with prior studies. C allele was regarded as a susceptible genetic marker for diabetic nephropathy [44,45] and progression of non-alcoholic steatohepatitis (NASH) [46].

We observed that some metals interact with EGFR and TNF-α SNPs, imposing an effect on the prevalence of MetS. The normal values of cobalt in plasma are 0.1–0.6 μg/L in the general population, and concentrations greater than 1 μg/L may indicate possible environmental or occupational exposure [47]. We proposed that the individuals carrying EGFR rs11977388 CT genotype may have a higher prevalence of MetS under an excessive amount of Co exposure. The average plasma Zn concentration is 700–1000 μg/L in healthy people [48]. The correlation between Zn and MetS was equivocal. Zn plays a vital role in the regulation of insulin, and high Zn levels may increase TG and pro-inflammatory cytokines [6,10]. Our result revealed that the individuals with EGFR rs3735061 AG genotype may have an elevated risk of MetS under too much Zn exposure. Se is an important essential element, and the reference value of plasma Se concentration is 40 to 200 μg/L in healthy adults [49]. Adequate Se concentration has antioxidative and cadioprotective properties, but a high dosage may impair glucose metabolism and increase blood pressure, even promoting MetS [9]. Our finding showed that the heterozygous genotypes of EGFR rs11977660 T > C, rs3823585 C > G, and rs3735061 G > A appeared to be more susceptible to higher plasma Se concentration, while TNF-α rs1800610 AA genotype played a protective role. In prior studies, TNF-α rs1800610 GA and AA genotypes were associated with a reduced risk in hepatocellular carcinoma [50], and AA genotype was observed to protect against rheumatoid arthritis [51], so we supposed that TNF-α rs1800610 AA might be a protective genotype in some pathogeneses. Pb was a toxic metal, but its association with MetS was not consistent [6,8]. We supposed that the results may vary by ethnicity and genetic inheritance. Our research showed that EGFR rs2472520 CG genotype was associated with a reduced MetS risk under higher plasma Pb concentration.

We only found one study investigating the relationship between genetic polymorphisms and MetS under occupational exposure to As, Cd, and Pb [52]. However, the selected genes and population were different from ours. Our research is the first one to analyze the interactive effects of EGFR/TNF-α SNPs and metals on the prevalence of MetS. The large sample size from both the general and occupational population is our strength. Lack of data of exercise and cross-sectional study are two of the limitations of our research. Furthermore, only rs2472520 CG x plasma Pb remained significant after Bonferroni correction. Nevertheless, we built 174 logistic regression models to thoroughly explore the complex interactions between 6 metals and 29 SNPs. We regressed one SNP in one model, and each logistic regression model was independent. Therefore, we considered that multiple testing may not be absolutely necessary in our study, but the stringent analysis did lead to a more powerful result. We proposed that rs2472520 CG was a protective genotype under higher plasma Pb level, which was the most convincing result in our research. Irrespective of the mild decrease in the statistical power, we still considered that other findings were of great value in this pioneering research. Further large-scale and long-term follow-up studies are warranted to illustrate the complicated interaction of multiple metals and genetic polymorphisms on MetS.

On the other hand, healthy worker effect could lead to selecting stronger workers in metal industry [53]. With increasing age, their BMI would be larger. The ratio of obesity (BMI ≥ 27) in our metal industrial workers was 26.6%, and this may be the reason why metal workers suffered from MetS more. MetS is a prodromal stage of CVD and chronic illness, and they have no serious health problems at the stage of MetS, so there is no influence on their work performance. In addition, 26 individuals (6.9%) of our metal industrial workers had diabetes, with a higher ratio than that of non-metal workers, but it was still in line with the average prevalence of diabetes in Taiwan [54]. With regard to hypertension, there is no significant difference in these two groups.

## 5. Conclusions

Environmental or occupational exposure to metals may influence the risk of MetS, and the effects may be modified by EGFR or TNF-α gene polymorphisms. Our study demonstrated some susceptible and protective EGFR and TNF-α genotypes under excessive exposure to cobalt, zinc, selenium, and lead. The most convincing result after performing Bonferroni correction was that EGFR rs2472520 CG genotype played a protective role under an excessive exposure to lead; furthermore, the other findings about susceptible genotypes were worthy of attention. It is urgent to find the individuals with susceptible genotypes and educate them to reduce the risk of MetS. We may provide novel insights for preventive and therapeutic strategies of MetS in the field of personalized medicine in the future.

## Figures and Tables

**Table 1:** Demographic characteristics, physical and biochemical data, and plasma metal concentrations in the participants with and without MetS.

Variable	Total *n* = 1015	Non-MetS *n* = 834	MetS *n* = 181	*p* Value
Worker group				<0.001
Metal workers	376 (37.0)	276 (33.1)	100 (55.2)	
Non-metal workers	639 (63.0)	558 (66.9)	81 (44.8)	
Gender				<0.001
Male	520 (51.2)	402 (48.2)	118 (65.2)	
Female	495 (48.8)	432 (51.8)	63 (34.8)	
Smoking	213 (21.0)	150 (18.0)	63 (34.8)	<0.001
Drinking alcohol	37 (3.7)	27 (3.3)	10 (5.7)	0.096
Age (year)	43.76 ± 10.11	43.25 ± 10.07	46.07 ± 9.98	0.001
BMI (kg/m^2^)	24.41 ± 3.96	23.62 ± 3.33	28.09 ± 4.51	<0.001
WC (cm)	81.72 ± 11.19	79.47 ± 9.88	92.20 ± 11.01	<0.001
SBP (mmHg)	117.98 ± 16.68	115.52 ± 15.55	129.29 ± 17.07	<0.001
DBP (mmHg)	72.19 ± 11.45	70.84 ± 10.97	78.41 ± 11.60	<0.001
Sugar (mg/dL)	94.52 ± 25.39	90.58 ± 15.01	112.68 ± 46.74	<0.001
TG (mg/dL)	126.55 ± 117.25	101.38 ± 62.14	242.55 ± 207.63	<0.001
HDL-C (mg/dL)	51.59 ± 13.91	54.30 ± 13.15	39.10 ± 9.93	<0.001
TC (mg/dL)	202.24 ± 37.33	200.00 ± 36.39	212.59 ± 39.87	<0.001
Uric acid (mg/dL)	5.76 ± 1.55	5.59 ± 1.49	6.47 ± 1.58	<0.001
ALT (IU/L)	26.16 ± 19.63	24.05 ± 18.49	35.90 ± 21.77	<0.001
Creatinine (mg/dL)	0.77 ± 0.18	0.77 ± 0.18	0.81 ± 0.17	0.003
Co (μg/L)	0.85 ± 0.29	0.85 ± 0.30	0.87 ± 0.25	0.286
Cu (μg/L)	1001.97 ± 270.83	992.99 ± 270.19	1043.74 ± 270.63	0.023
Zn (μg/L)	849.68 ± 275.77	840.16 ± 277.27	893.98 ± 264.95	0.018
Se (μg/L)	207.54 ± 107.33	198.82 ± 102.99	247.69 ± 117.62	<0.001
As (μg/L)	6.07 ± 8.07	5.99 ± 8.63	6.47 ± 4.65	0.463
Pb (μg/L)	0.33 ± 0.52	0.30 ± 0.49	0.48 ± 0.63	<0.001

Data are presented as n(%) or mean ± standard deviation. MetS—metabolic syndrome; BMI—body mass index; WC—waist circumference; SBP—systolic blood pressure; DBP—diastolic blood pressure; TG—triglyceride; HDL-C—high-density lipoprotein cholesterol; TC—total cholesterol; ALT—alanine aminotransferase.

## Supplementary Materials

The following are available online at https://www.mdpi.com/article/10.3390/toxics9090225/s1, Table S1: title, Genotyping of EGFR and TNF-α SNPs by TWB1 and TWB2 chips Table S2: Demographic characteristics, physical and biochemical data, and plasma metal concentrations of two groups of participants.
