# Supplementary material for: The Relationship between Metabolic Syndrome and Plasma Metals Modified by EGFR and TNF-α Gene Polymorphisms
Source: Toxics. 2021 Sep 16;9(9):225. doi: 10.3390/toxics9090225 (PMC8473312; doi:10.3390/toxics9090225)
Supplement: Supplementary file 1 [file toxics-09-00225-s001.zip › Toxics-1344024-supplementary materials.pdf]

toxics

MDPI

Article

The Relationship between Metabolic Syndrome and Plasma Metals Modified by EGFR and TNF-α Gene Polymorphisms

Tzu-Hua Chen, Wei-Shyang Kung, Hung-Yu Sun, Joh-Jong Huang, Jia-Yi Lu, Kuei-Hau Luo and Hung-Yi Chuang

Table S2. Demographic characteristics, physical and biochemical data, and plasma metal concentrations of two groups of participants.

Variable	Total<br>n = 1015	Non-metal workers<br>n = 639	Metal workers<br>n = 376	*p* value
MetS	181 (17.8)	81 (12.7)	100 (26.6)	<0.001
Gender				0.057
Male	520 (51.2)	340 (53.2)	180 (47.9)	
Female	495 (48.8)	299 (46.8)	196 (52.1)	
Smoking	213 (21.0)	90 (14.1)	123 (32.7)	<0.001
Drinking alcohol	37 (3.7)	30 (4.8)	7 (1.9)	0.011
Age (year)	43.76 ± 10.11	43.50 ± 9.65	44.18 ± 10.83	0.301
BMI (kg/m^2)	24.41 ± 3.96	24.04 ± 3.45	25.05 ± 4.62	<0.001
WC (cm)	81.72 ± 11.19	81.98 ± 10.78	81.30 ± 11.86	0.351
SBP (mmHg)	117.98 ± 16.68	115.30 ± 16.37	122.53 ± 16.23	<0.001
DBP (mmHg)	72.19 ± 11.45	73.01 ± 11.16	70.79 ± 11.81	0.003
Sugar (mg/dL)	94.52 ± 25.39	92.65 ± 17.86	97.70 ± 34.41	0.002
TG (mg/dL)	126.55 ± 117.25	117.76 ± 93.32	141.49 ± 148.33	0.002
HDL-C (mg/dL)	51.59 ± 13.91	55.10±13.69	45.62 ± 12.15	<0.001
TC (mg/dL)	202.24 ± 37.33	198.15 ± 35.36	209.20 ± 39.54	<0.001
Uric acid (mg/dL)	5.76 ± 1.55	5.75 ± 1.57	5.76 ± 1.52	0.927
ALT (IU/L)	26.16 ± 19.63	24.93 ± 18.99	28.26 ± 20.53	0.009
Creatinine (mg/dL)	0.77 ± 0.18	0.79 ± 0.18	0.75 ± 0.16	0.001
Co (μg/L)	0.85 ± 0.29	0.78 ± 0.29	0.97 ± 0.26	<0.001
Cu (μg/L)	1001.97 ± 270.83	950.29 ± 265.75	1088.93 ± 257.00	<0.001
Zn (μg/L)	849.68 ± 275.77	791.36 ± 258.86	947.82 ± 275.82	<0.001
Se (μg/L)	207.54 ± 107.33	146.12 ± 46.65	311.91 ± 100.48	<0.001
As (μg/L)	6.07 ± 8.07	4.76 ± 3.35	8.31 ± 12.20	<0.001
Pb (μg/L)	0.33 ± 0.52	0.05 ± 0.04	0.83 ± 0.58	<0.001

Data are presented as n(%) or mean ± standard deviation. MetS—metabolic syndrome; BMI—body mass index; WC—waist circumference; SBP—systolic blood pressure; DBP—diastolic blood pressure; TG—triglyceride; HDL-C—high-density lipoprotein cholesterol; TC—total cholesterol; ALT—alanine aminotransferase.
